# Synergistic Antifungal Effects of Honokiol and Fluconazole Against Oral *Candida*: Implications for Managing Drug‐Resistant Infections

**DOI:** 10.1002/cre2.70251

**Published:** 2026-01-29

**Authors:** Maribasappa Karched, Mohammad Irshad, Jawad M. Behbehani

**Affiliations:** ^1^ Oral Microbiology Laboratory, Department of Bioclinical Sciences, College of Dentistry Kuwait University Kuwait City Kuwait; ^2^ Dasman Diabetes Institute Kuwait City Kuwait; ^3^ Department of Restorative Sciences, College of Dentistry Kuwait University Kuwait City Kuwait

## Abstract

**Objectives:**

Antifungal drug resistance poses a major challenge in treating oral *Candida* infections. This study aimed to evaluate the antifungal activity of Honokiol, alone and combined with Fluconazole, against oral *Candida* isolates, and to investigate its mechanism of action via ergosterol biosynthesis inhibition and cell wall disruption.

**Material and Methods:**

Susceptibility testing was performed using CLSI (M27‐A3) methodology on 16 oral *Candida* isolates, 8 endodontic isolates, and the reference strain ATCC 24433. Minimum inhibitory concentrations (MICs) of Honokiol and Fluconazole were determined alone and in combination. Ergosterol inhibitory assays and scanning electron microscopy (SEM) were used to assess cell wall integrity and morphological changes.

**Results:**

Honokiol exhibited MICs of 16–64 µg/mL (oral isolates), 16 µg/mL (endodontic isolates), and 32 µg/mL (ATCC 24433). In combination with Fluconazole, Honokiol's MICs decreased 4‐fold (4–16 µg/mL), while Fluconazole's MICs dropped 2‐ to 4‐fold (1–32 µg/mL). Synergy was confirmed by a 95.61% reduction in fungal growth (OD600) compared to controls. SEM revealed severe cell wall distortion, rupture, and cytoplasmic leakage. Honokiol significantly inhibited ergosterol biosynthesis, disrupting cellular integrity.

**Conclusion:**

Honokiol demonstrates potent antifungal activity against oral *Candida* isolates by targeting ergosterol biosynthesis and compromising cell wall integrity. Its synergistic enhancement of Fluconazole's effect suggests clinical potential as an adjunct therapy, potentially reducing resistance and lowering required drug doses in oral and endodontic candidiasis.

## 1 Introduction

*Candida albicans* is a commensal fungus present in approximately 50% of the global population's oral cavities (Aminzadeh et al. 2016; Calderone and Fonzi 2001; Cui et al. 2013). Clinical samples from a dental clinic in Kuwait revealed a variety of *Candida* species in oral cavity, with *C. albicans* being the most prevalent (Behbehani et al. 2017). However, its overgrowth can lead to oral infections, commonly referred to as candidiasis (Lu 2021). This organism poses a significant risk for severe infections in immunocompromised individuals or those with altered natural flora (Ohshima et al. 2018). *C. albicans* readily grows in the oral cavity, particularly on the tongue, in periodontal pockets, and on mucosal surfaces, and it can invade underlying tissues (Lu 2021; Ohshima et al. 2018). Additionally, *C. albicans* can infiltrate the root canal system through dental caries, presenting significant challenges during patient treatment (Shah et al. 2016). The persistence of *C. albicans* in the root canal system has been identified as a leading cause of endodontic treatment failures (Ahmed et al. 2024; Kumar et al. 2015). In clinical settings, various root canal irrigant solutions are employed to eliminate infections; however, certain fungi, including *C. albicans*, exhibit resistance to the antibacterial agents in these solutions (Kumar et al. 2015). Furthermore, the frequent use of these irrigants raises serious clinical concerns due to their toxicity to periapical tissues, potential damage to permanent tooth follicles, and risk of acute allergic reactions (Mohammadi 2008).

Fluconazole is commonly utilized as the primary first‐line treatment for oral *Candida* infections (Garcia‐Cuesta et al. 2014; Madane et al. 2022). The clinicians are also recommending topical antifungal drugs such as nystatin, miconazole, clotrimazole, and ketoconazole. However, the indiscriminate use of these medications has led to the emergence of resistance against many antifungal drugs (Wisplinghoff et al. 2014). Amphotericin B, recognized as the gold standard antifungal, is limited in clinical application due to its nephrotoxicity and other adverse effects on patients (Jensen et al. 2015). This situation underscores the necessity for safe, innovative, and effective antifungal compounds.

The predominant sources of bioactive compounds utilized in medicine and therapy have historically been derived from natural origins (Newman and Cragg 2020). In recent years, the dental care literature has increasingly focused on natural therapeutic products in conjunction with commercially synthesized alternatives (Moghadam et al. 2020). Natural compounds are increasingly preferred over synthetic alternatives due to their superior safety profiles, biocompatibility, and a wide range of biological activities. In recent years, many herbal formulation‐based oral rinses have demonstrated strong antifungal properties against *C. albicans*, competing with and even exceeding the effectiveness of commercial rinses at elevated concentrations (Safiya et al. 2024).

Numerous studies have highlighted the antifungal and antimicrobial properties of phytochemicals derived from Magnolia species (Cristea et al. 2024; Yan et al. 2020). Honokiol, a prominent active compound is found in all parts of Magnolia species. It is a polyphenolic compound (5,5′‐diallyl‐2,4′‐dihydroxybiphenyl) that belongs to the neolignan biphenols class. As a polyphenol, it is relatively small and capable of interacting with cell membrane proteins through intermolecular interactions, including hydrogen bonding, hydrophobic interactions, and aromatic pi orbital co‐valency (Woodbury et al. 2013). Various pharmacological properties of Honokiol have been documented, including antimicrobial (Rauf et al. 2021; Sun et al. 2024), antifungal (Yan et al. 2020), analgesic (Lin et al. 2009), antithrombotic (Hu et al. 2005), anti‐tumorigenic, and neuroprotective effects (Woodbury et al. 2013). In traditional medication, topical preparation of Honokiol used a safe and effective way for treating skin diseases (Li et al. 2022). The administration of Honokiol microemulsion at doses up to 500 µg/kg has been shown to be nontoxic in Sprague–Dawley rats (Zhang et al. 2015).

Despite the reported antimicrobial and antifungal activities of Honokiol, there is limited information regarding its synergistic effects with azole drugs. This study examines the antifungal properties of Honokiol against oral and endodontic *C. albicans*, utilizing a range of methodologies to evaluate its fungicidal effect. Additionally, the study investigates the synergistic effects of Honokiol when combined with Fluconazole on the susceptibility of *C. albicans*. The integration of these two antifungal agents may offer a promising approach for addressing the proliferation of Candida species in future therapeutic strategies.

## 2 Materials and Methods

### 2.1 Chemicals and Sample Collection

Honokiol (with a purity of ≥ 95%), Fluconazole, and the constituents of the culture medium, were obtained from Sigma‐Aldrich, USA. Oral *Candida* species samples were obtained from dental patients at the Kuwait University Dental Clinic (KUDC) utilizing a standard oral rinse method (Samaranayake et al. 1986). The samples were identified using CHROMagar medium (CHROMagar Candida, France) and VITEK‐2 system assays (bioMerieux, Craponne, France). Endodontic isolates and the ATCC strain were obtained from the Oral Microbiology Laboratory at the College of Dentistry, Kuwait University, Kuwait. While all strains were included in the MIC experiments, reference strains and clinical isolates used in different experiments are mentioned in figure legends.

### 2.2 Minimum Inhibitory Concentration

The method outlined by the Clinical Laboratory Standards Institute (CLSI) for yeasts (M27‐A3) was used to determine the minimum inhibitory concentration (MIC) of the test compound and the drug (Wayne 2008), as we described in earlier study (Behbehani et al. 2023). The MIC was assessed in RPMI‐164 medium (Cat no. R6504, Sigma‐Aldrich, USA), which was buffered to a pH of 7.0 using 0.165 M morpholinepropanesulfonic acid. An overnight culture of *Candida* cells was diluted in the medium, and a 100 μL aliquot of this diluted inoculum was added to each well of a 96‐well U‐bottom tissue culture plate, achieving a final inoculum of approximately 2.5 × 10^3^ cells/mL. The concentration range tested for Honokiol was from 1 to 512 µg/mL, while Fluconazole ranged from 0.50 to 128 µg/mL. The medium without drugs served as the control, and a blank control containing only the medium was also included. The plates were incubated at 37°C for 48 h. After incubation, the plates were examined visually, and the MIC was defined as the lowest concentration of the test agents that inhibited visible growth compared to the growth control. All assays were conducted in triplicate.

### 2.3 Synergistic Activity of Honokiol and Fluconazole

Synergistic activity was assessed using the checkerboard microtiter plate method (Odds 2003; Wayne 2008), as described in our earlier research (Behbehani et al. 2019). Honokiol and Fluconazole were serially diluted in RPMI in various combinations. The final concentrations for Honokiol ranged from 4 to 256 µg/mL, while for Fluconazole, they ranged from 1 to 64 µg/mL. Candida cells were introduced into each well at a final concentration of approximately 2.5 × 10^3^ cells/mL, followed by incubation for 48 h at 37°C. The growth of Candida cells was evaluated visually, and all assays were conducted in triplicate.

The fractional inhibitory concentration index (FICI) was utilized to assess the interactions between Honokiol and the drugs. The formula for FICI is: FICI* =* MIC (Honokiol with Fluconazole)/MIC (Honokiol alone) + MIC (Fluconazole with Honokiol)/MIC (Fluconazole alone). A FICI value of ≤ 0.5 indicates a synergistic interaction between Honokiol and Fluconazole, a value between 0.5 and 4 suggests no interaction, and a value > 4 indicates an antagonistic interaction between the two agents (Behbehani et al. 2019).

### 2.4 Synergistic Effect of Honokiol and Fluconazole Drug on the Growth of *C. albicans*

The synergistic effect of both molecules on growth of *C. albicans* was examined (Behbehani et al. 2019). Fresh overnight growth of candia calls (approximately 1 × 10^6^ cells, optical density A595* =* 0.1) was inoculated into YPD medium. The MIC and sub‐MIC values of Honokiol alone or Honokiol with Fluconazole drug was added to the medium. The cells were incubated at 37°C and 120 rpm, using IKA KS 3000 Shaking Incubator (Staufen, Germany). The optical density of cells was recorded per hours by using Eppendorf Spectrophotometer (Hamburg, Germany).

### 2.5 Synergistic Effect of Honokiol and Fluconazole Drug on Ergosterol Biosynthesis

The intracellular sterols were extracted using a standard procedure (Breivik and Owades 1957), as outlined in our earlier research (Irshad et al. 2013). A Candida colony from an overnight culture on a Sabouraud dextrose agar plate was used to inoculate 10 mL of Sabouraud dextrose broth. The MIC and sub‐MIC values of Honokiol alone or Honokiol with Fluconazole drug were added to the broth. The cultures were incubated for 16 h at 37°C and then Candida cells were collected by centrifugation at 4000× *g* for 5 min. The weight of the cell pellet was measured. To each pellet, 3 mL of a 25% ethanolic potassium hydroxide solution was added and vortexed for 3 min. The resulting cell suspensions were transferred to sterile borosilicate glass screw‐cap tubes and incubated at 85°C in a water bath for 1 h, then allowed to cool to room temperature. Sterols were extracted by adding a mixture of 1 mL of sterile Milli‐Q water and 3 mL of n‐heptane, followed by vigorous vortex mixing for 3 min. The n‐heptane layer was then transferred to a clean borosilicate glass screw‐cap tube and stored at −20°C.

The concentration of sterols was measured spectrophotometrically between 230 and 300 nm using a spectrophotometer (Eppendorf, USA). The ergosterol content was calculated as a percentage of the wet weight of the cells using the following formulas:

%Ergosterol+%24(28)dehydroergosterol(DHE)=[(A281.5/290)/pelletweight]

%24(28)DHE=[(A230/518)/pelletweight]

%ergosterol=[%ergosterol+%24(28)DHE]/[%24(28)DHE]

The values of 290 and 518 represent the *E* values (in percentages per centimeter) determined for crystalline ergosterol and 24 (28) DHE, respectively.

### 2.6 Biofilm Inhibition Assay

A standardized method was employed in this study (Behbehani et al. 2019). Mature Candida biofilms were developed in the wells of polystyrene 96‐well microtiter plates (Corning Inc., Corning, NY, USA). The MIC of Honokiol was prepared in RPMI 1640 medium and introduced into each well (200 µL/well), followed by incubation for 24 h at 37°C. After incubation, the medium was removed, and the wells were rinsed gently with sterile PBS (pH 7). The biofilm density was examined using confocal laser microscopy (Zeiss LSM 500) with Cyto‐9 fluorescence dye. The biofilm viability was assessed through the MTT assay, and the percentage of biofilm inhibition was determined. All experiments were conducted in triplicate.

### 2.7 Effect of Honokiol on the Biofilm of Dentine Substrate

For this study, we used the standard method (Behbehani et al. 2023). Freshly extracted teeth were obtained from the Kuwait Dental clinic. The pulp chamber of the teeth was opened using a cutting machine (Accutom‐100, USA). The teeth slice underwent washing and sterilization through treatment with 17% EDTA, followed by 2.5% sodium hypochlorite for 4 min, and then air‐dried for 10 min. The teeth slice was then immersed in a freshly prepared suspension of *C. albicans* cells (1 × 106) in YEPD medium and incubated with shaking at 120 rpm at 37°C for 24 h. Following the incubation, the teeth slice was moved to fresh medium containing the test molecule (MIC) and re‐incubated for another 24 h. At the conclusion of the treatment period, the dental slice was carefully washed three times with PBS and prepared for scanning electron microscopy (SEM) (JEOL, Carryscope JCM 5700).

### 2.8 Study of Hyphae Formation and Cells Morphology

A sample of overnight culture cells, approximately 10^6^ cells/mL, was introduced into YPD medium enriched with 10% fetal bovine serum (FBS) (Sun et al. 2015). The MIC of Honokiol was incorporated into the medium and maintained at 37°C under the 20× objective lens of the Cell Observer Microscope (Zeiss LSM). The morphological alterations of Candida cells were recorded live every 15 min. Untreated cells served as a positive control.

### 2.9 Confocal Scanning Laser Microscopy

The impact of Honokiol on the membranes of *Candida* cells was evaluated using the fluorescent dye Propidium iodide (PI) (Behbehani et al. 2017). Approximately 1 × 10^6^ *Candida* cells were placed in a YPD medium and treated with Honokiol at 37°C while being continuously shaken at 120 rpm for 12 h. To verify the permeabilization of the cell membranes, 1 µg/mL of the dye was introduced, and the cell suspensions were incubated again at 37°C for an additional 30 min. The uptake of the dye was observed using confocal microscopy (Zeiss LSM 500).

### 2.10 Statistical Analysis

The experiments were conducted three times. Statistical evaluations were carried out using SPSS vs 23 software (SPSS Inc., USA). The differences between the groups were assessed using a *t*‐test. A *p*‐value of 0.05 or less was deemed statistically significant.

## 3 Results

### 3.1 Minimum Inhibitory Concentration

Honokiol exhibited significant antifungal activity against both clinical isolates and the ATCC strain of *C. albicans*. The MICs of Honokiol for the 16 oral isolates ranged between 16 and 64 µg/mL, while for the 8 endodontic isolates was 16 µg/mL. The MIC for the ATCC 24433 strain was 32 µg/mL. The MICs ranges for Fluconazole for the oral isolates ranged between 4 and 128 µg/mL, for the endodontic isolates ranged between 16 and 128 µg/mL, and for the ATCC 24433 strain was 64 µg/mL (Table 1).

**Table 1:** Minimum inhibitory concentration of Honokiol alone and in combination with Fluconazole against *C. albicans*.

	Honokiol		Fluconazole		Σ FICI
	MIC (µg/mL)		MIC (µg/mL)		
*C. albicans*	Alone/Combo	FICI	Alone/Combo	FICI	
ATCC 24433	32/8	0.250	64/8	0.125	0.375
ENDO 902	16/4	0.250	64/4	0.063	0.313
ENDO 903	16/8	0.500	64/16	0.250	0.750
ENDO 904	16/4	0.250	128/8	0.063	0.313
ENDO 905	16/4	0.250	64/4	0.063	0.313
ENDO 906	16/8	0.500	128/16	0.125	0.625
ENDO 908	16/4	0.250	32/4	0.125	0.375
ENDO 910	16/4	0.250	16/4	0.250	0.500
ENDO 911	16/4	0.250	128/16	0.125	0.375
ORS 1	32/8	0.250	32/4	0.125	0.375
ORS 3	16/4	0.250	64/8	0.125	0.375
ORS 4	64/16	0.250	128/16	0.125	0.375
ORS 8	32/8	0.250	8/2	0.250	0.500
ORS 11	64/16	0.250	128/16	0.125	0.375
ORS 13	32/8	0.250	128/8	0.063	0.313
ORS 15	64/16	0.250	64/8	0.125	0.375
ORS 16	64/16	0.250	128/32	0.250	0.500
ORS 17	16/8	0.500	8/4	0.500	1.000
ORS 18	16/4	0.250	8/2	0.250	0.500
ORS 19	16/4	0.250	16/2	0.125	0.375
ORS 20	16/8	0.500	16/4	0.250	0.750
ORS 25	16/4	0.250	64/8	0.125	0.375
ORS 26	16/4	0.250	4/1	0.250	0.500
ORS 29	16/8	0.500	32/8	0.250	0.750
ORS 30	16/4	0.250	32/8	0.250	0.500

Abbreviations: ATCC, American type culture collection; Combo, combination of Honokiol and Fluconazole; Endo, endodontic isolates; FICI, fractional inhibitory concentration index; MIC, minimum inhibitory concentration; ORS, oral isolates.

### 3.2 Synergistic Activity of Honokiol With Fluconazole

Different combinations of Honokiol and Fluconazole were systematically evaluated for their synergistic effects against the oral and endodontic isolates. The combination of Honokiol with Fluconazole resulted in a significant reduction in the MIC values compared to the individual administration of each drug (Table 1). The MICs for Fluconazole decreased markedly from a range of 4–128 µg/mL to 1–32 µg/mL, while the MICs for Honokiol was significantly reduced from 16–64 µg/mL to 4–16 µg/mL (*p* < 0.01). The synergistic effects of the Honokiol and Fluconazole combination were confirmed (FICI of ≤ 0.50) in the ATCC 24433 strain, 6 endodontic isolates, and 13 oral isolates. None of the samples showed the antagonistic interaction between the two agents (Table 1).

### 3.3 Synergistic Effect on *Candida* Cell Growth

**Figure 1:**
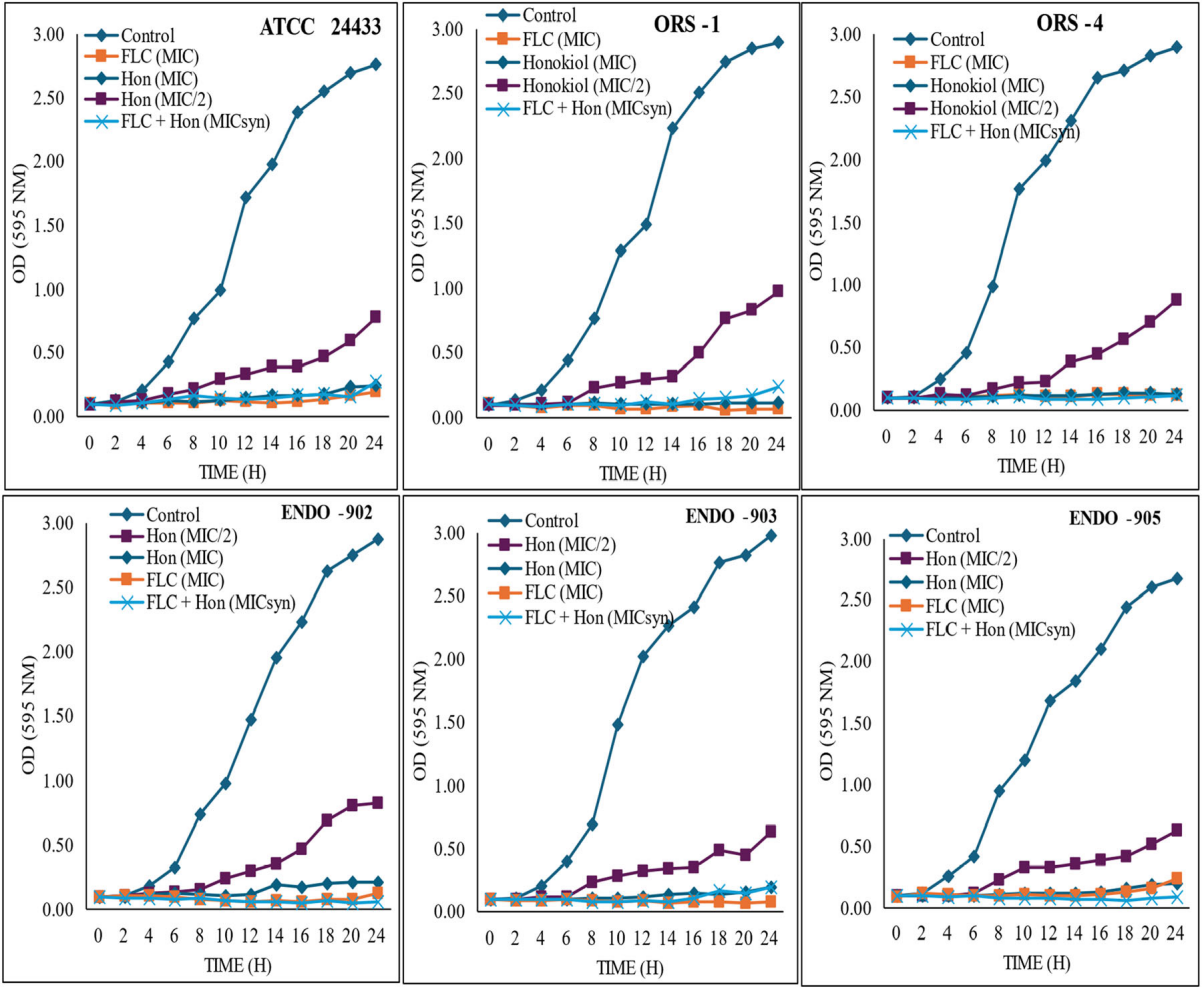
Presents the impact of Honokiol (Hon) both alone and in conjunction with Fluconazole (FLC) on the growth of *C. albicans*. The graph depicts the growth curves of *C. albicans* strains (ATCC 24433, ORS‐1 and ‐4, and ENDO‐902, ‐903, and ‐905) in the presence of Hon (MIC and sub‐MIC), as well as in a synergistic combination with FLC (MIC syn). The growth curves are plotted based on optical density at 595 nm over time (h).

The growth curve has been plotted by recording time versus absorbance at 595 nm. Figure 1 shows the effect of MIC, sub‐MIC values of Honokiol and combination with Fluconazole (MICsyn) on the growth pattern of *C. albicans* oral (ORS 1 and 4) and endodontic (ENDO‐902, ‐903, and ‐905) isolates and ATCC 24433 strain, respectively. Untreated *Candida* cells (control) showed a normal pattern of growth with a lag phase of 0–2 h, an active exponential phase of 8–12 h before attaining stationary phase. The increase in the concentration of Honokiol leads to a significant decrease in growth with suppressed and delayed exponential phases. Complete cessation of growth was observed at MIC and MICsyn values. The average decrease in growth of six *C. albicans* (after 24 h) at MIC of Honokiol and Fluconazole and MICsyn, measured as a change in optical density with respect to control was 96.14%, 95.25%, and 95.61%, respectively.

### 3.4 Synergistic Effect on Ergosterol Synthesis in *Candida* Cells

**Figure 2:**
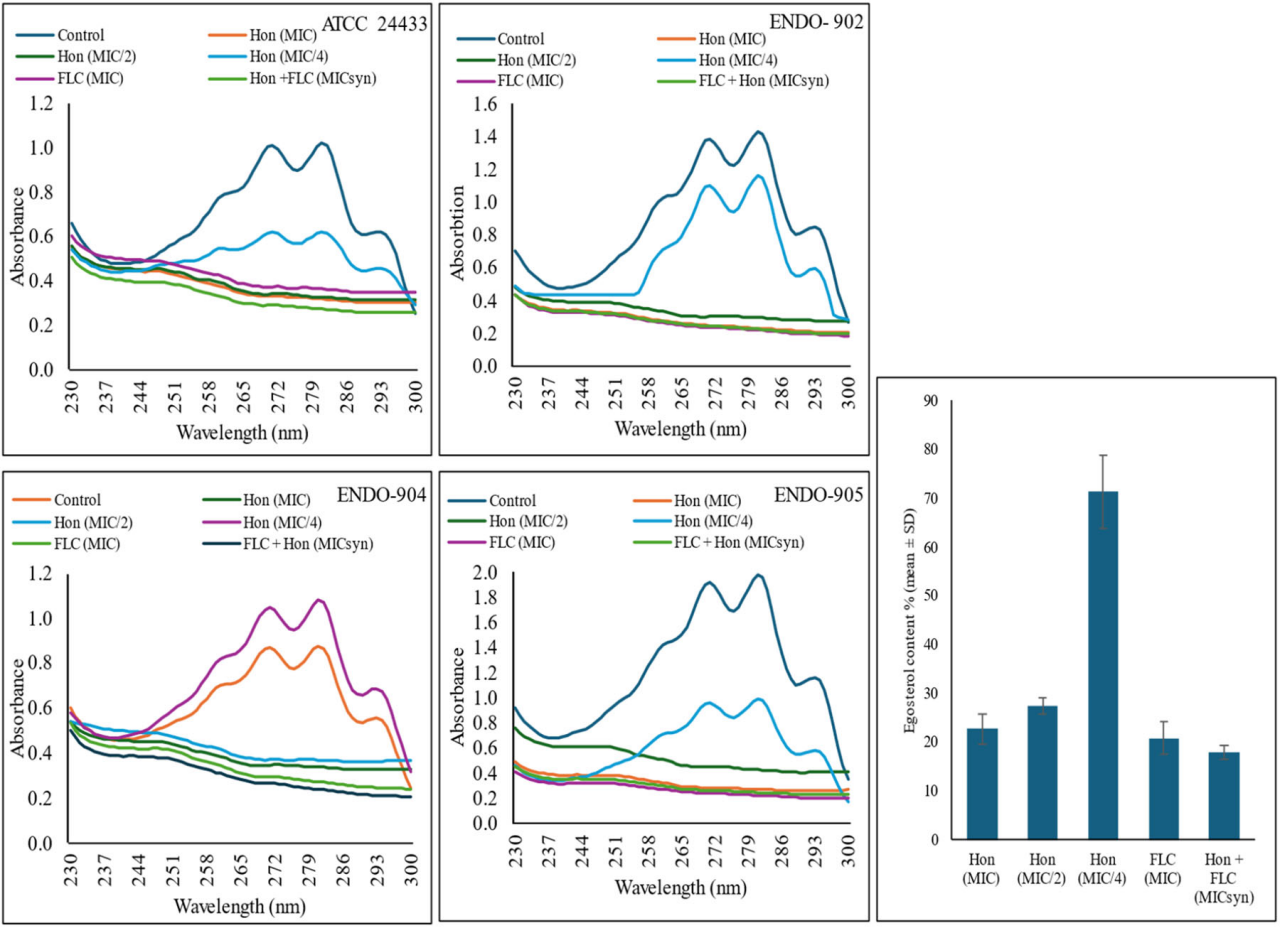
Spectrophotometric sterol profiles of *C. albicans* (ATCC 24433, and ENDO‐902, ‐904, and ‐905) in the presence of Hon (MIC and sub‐MIC), as well as in a synergistic combination with FLC (MIC syn). Sterols were extracted from the cells and spectral profiles between 230 and 300 nm were determined.

Ergosterol levels in the membranes of both treated and untreated *C. albicans* cells were measured. Figure 2 demonstrates a marked reduction in ergosterol levels in cells treated with Honokiol (MIC), Fluconazole (MIC), and a combination of both (MICsyn), as evidenced by the flat curve (*p* < 0.05). However, the untreated control cells exhibited pronounced peaks of ergosterol. The average reductions in ergosterol content for cells treated with MIC/4, MIC/2, MIC of Honokiol, MIC of Fluconazole, and MICsyn of Honokiol and Fluconazole were 28.7%, 72.6%, 77.3%, 79.1%, and 82.0%, respectively.

### 3.5 Biofilm Inhibition

**Figure 3:**
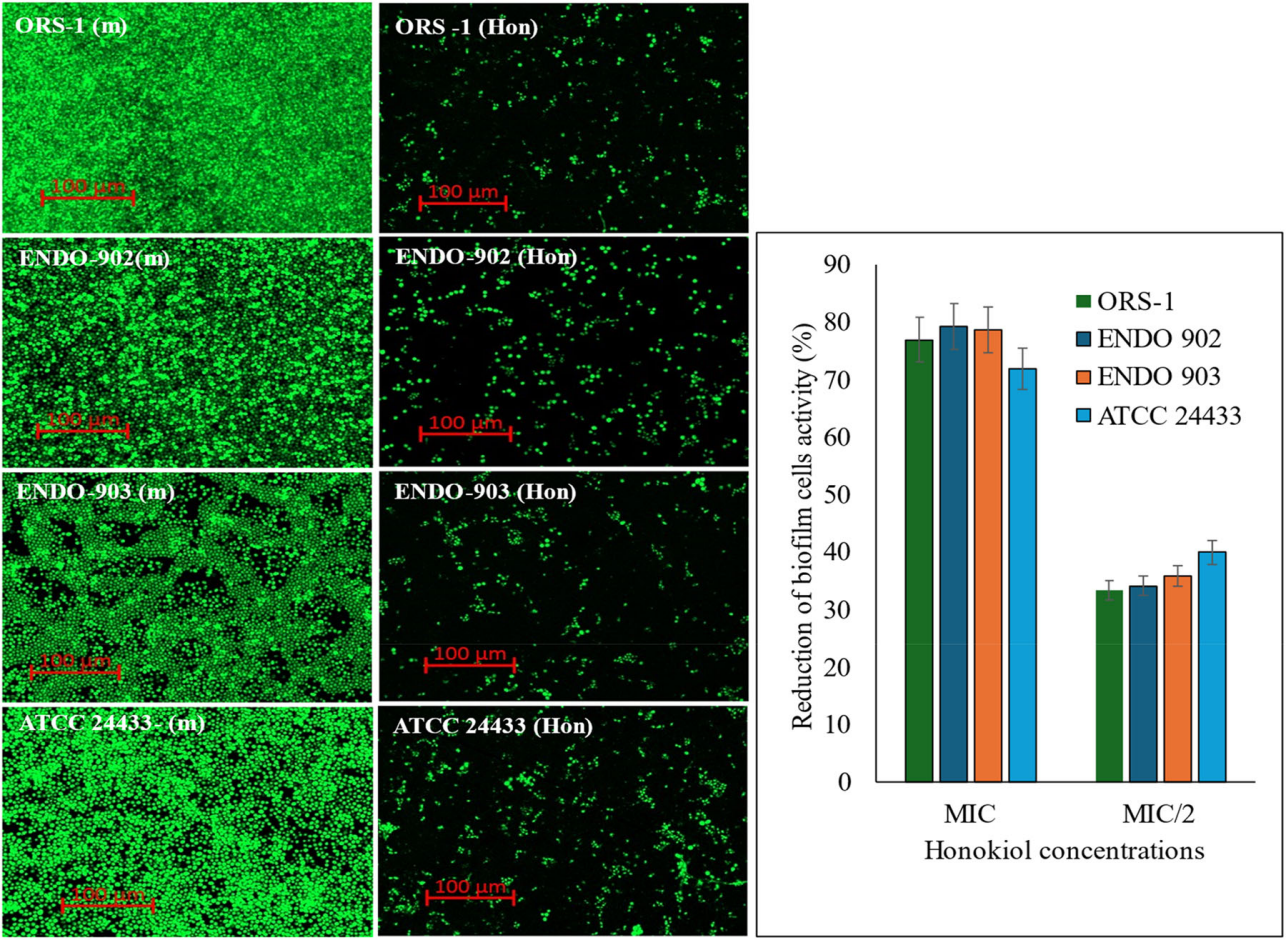
The left panel presents the effect of Honokiol (Hon) on the mature biofilm of *C. albicans* (m). The left panel showed a reduction in biofilm cell activity after treatment with Honokiol. The mean differences between the control and test molecules at the MIC level were statistically significant (*p* < 0.01). ENDO, endodontic isolates; MIC/2, half of the MIC concentration; ORS, oral isolates.

The effect of Honokiol (MIC) was assessed on established mature biofilms of *C. albicans*. Figure 3 presents a CLSM image of the mature biofilm on the left, while the right side depicts the biofilm structure posttreatment. The CLSM image distinctly demonstrates the eradication of the biofilm in the treated sample. The metabolic activity of the biofilm, evaluated through the MTT (3‐(4,5‐dimethylthiazol‐2‐yl)‐2,5‐diphenyltetrazolium bromide) reduction, indicated an average reduction of 35.9% at the MIC/2, whereas it was 76.8% at the MIC level. Our results indicate that Honokiol significantly inhibits the formation of mature biofilms (*p* < 0.01).

### 3.6 Inhibitory Activity on Hyphae Formation

**Figure 4:**
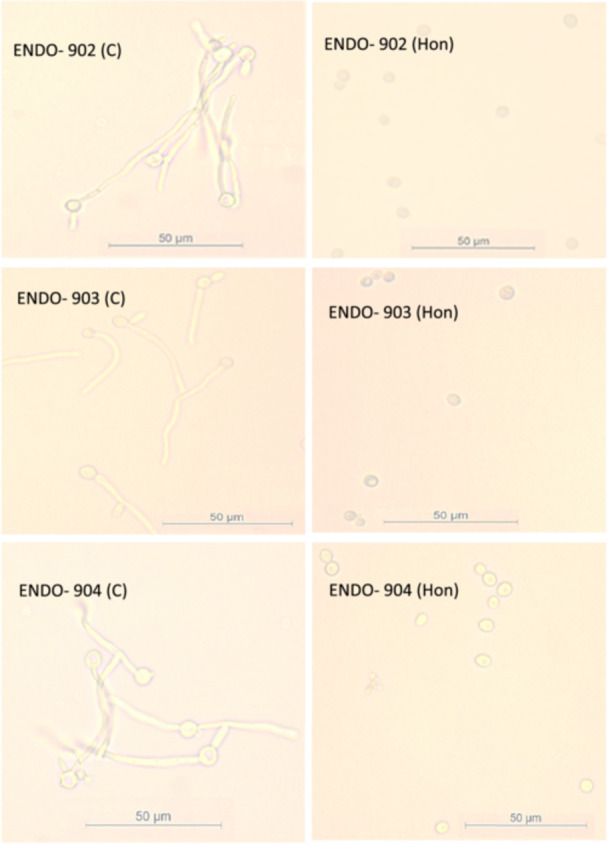
Effect of Honokiol on the hyphae growth of *C. albicans* (Endo‐902, ‐903, and ‐904). The live cells photograph was recorded by the cells observer microscope at a magnification of 20×. Photograph of both untreated (c) and treated cells (Hon) were taken at 3‐h time point, respectively.

The hyphae‐inducing medium was employed to assess the inhibitory effects of Honokiol on hyphae formation. Real‐time images of the cells transitioning from yeast to filamentous forms were captured using a Live Cell Observer microscope. In Figure 4, the left panel presents the untreated *C. albicans* (control) cells, while the right panel depicts the cell morphology posttreatment with Honokiol at the MIC. The untreated controls demonstrated significant hyphae growth after 3 h, whereas the presence of Honokiol (MIC) effectively inhibited any hyphae development. These findings clearly demonstrate that Honokiol is a potent inhibitor of hyphae growth.

### 3.7 Change of Cellular Morphology

**Figure 5:**
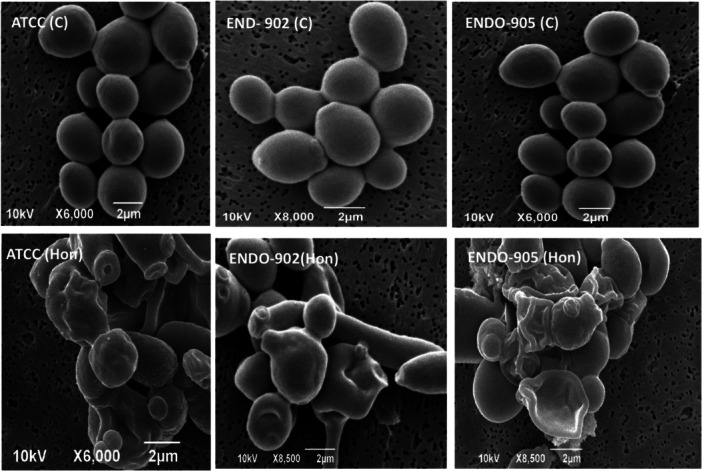
Scanning electron micrograph of *C. albicans* cells with and without treatment with Honokiol (MIC). The untreated *Candida* cells are labeled as ATCC (C), ENDO‐902 (C), and ENDO‐904 (C), while the treated cells with Honokiol are labeled as ATCC (Hon), ENDO‐902 (Hon), and ENDO‐905 (Hon).

Scanning electron micrographs demonstrated that all *Candida* strains studied adhered to dentine. *Candida* cells treated with Honokiol showed notable morphological changes. The micrograph illustrating *C. albicans* cells subjected to the MIC of Honokiol is depicted in Figure 5. In the SEM images, untreated *Candida* cells exhibited an oval shape with smooth surfaces and discernible polar bud scars. Conversely, cells treated with Honokiol at the MIC for 12 h displayed significant deformation, characterized by convoluted and irregular surfaces, along with the presence of lytic material in vesicular form. The treated cells underwent a complete transformation in morphology, evidenced by the emergence of deep furrows and wrinkles on their surfaces.

## 4 Discussion

The findings demonstrate the promising antifungal potential of Honokiol against *C. albicans*, as it targets multiple mechanisms essential for fungal survival and virulence. Honokiol exhibited significant antifungal activity, with MIC values ranging from 16 to 64 µg/mL across clinical isolates and the ATCC strain. These MIC values demonstrated that Honokiol exhibit comparable effect, particularly against isolates that are resistant or less sensitive to Fluconazole. Present finding is supported by a study, where *C. albicans* strains show MICs range of 16–32 μg/mL (Sun et al. 2015). Additionally, another study reported that the MIC of Honokiol against *C. auris* strains was 16 mg/L (Mares et al. 2024).

Another key finding of this study is the synergistic interaction between Honokiol and Fluconazole. Our study represents the first evidence of the combined effects of Honokiol and Fluconazole antifungal drugs against *C. albicans*. The combination significantly reduced the MIC values for both agents, with Fluconazole's MIC decreasing from 4–128 µg/mL to 1–32 g/mL and Honokiol's MIC decreasing from 16–64 µg/mL to 4–16 µg/mL, suggesting the compatibility of Honokiol with Fluconazole for combined therapeutic use. Particularly for those oral and endodontic isolates that have synergistic effects could enhance treatment effect and potentially lower the required doses of each agent, reducing side effects and minimizing the risk of resistance development. In the present study, many clinical isolates showed Fluconazole MIC values of 128 µg/mL underscoring the growing concern of antifungal resistance (Kessler et al. 2022). The combination of Fluconazole and Honokiol effect against these strains provides a potential alternative, particularly in cases where resistance to conventional antifungals limits treatment options. The synergistic effects of Honokiol with Fluconazole were further confirmed through the 24‐h growth study, and results showed a significant reduction in optical density (95.6%) compared to the control. The results demonstrated that *C. albicans* culture treated with Honokiol at MICs or in combination with Fluconazole at MICs were unable to be recovered.

The inhibition of ergosterol biosynthesis is a well‐established mechanism of action for antifungal agents, as ergosterol is an essential component of fungal cell membranes (Villasmil et al. 2020). Honokiol demonstrated a dose‐dependent reduction in ergosterol levels, as evidenced by a marked decrease in ergosterol peaks in treated cells compared to untreated controls. The synergistic effect on reduction in ergosterol content was also substantial, with decreases of 98.6% observed at MIC syn concentrations of Honokiol and Fluconazole. These findings suggest that Honokiol's antifungal action is mediated, at least in part, by disrupting ergosterol biosynthesis, compromising membrane integrity, and ultimately inhibiting fungal growth. A study also demonstrated that Honokiol inhibits ergosterol content, disturbs the expression of genes related to ergosterol biosynthesis and decreases Pma 1p H+ ‐ATPase activity, which resulted in the abnormal pH in vacuole and cytosol in *C. albicans* (Sun and Liao 2020).

We also found that the inhibitory effect of Honokiol on transition of *C. albicans* yeast to hyphae is another important antifungal property demonstrated by Honokiol. The transition from yeast to filamentous hyphal forms is a key virulence factor in *C. albicans*, facilitating tissue invasion and immune evasion (Chow et al. 2021). In a previous study, Honokiol inhibited hyphae growth on nutrient‐poor spider medium, liquid RPMI 1640, and GlcNAc medium, but not in FBS YPD medium (Sun et al. 2015). In the present study, real‐time imaging using a Live Cell Observer microscope revealed that Honokiol effectively prevented hyphae growth in a hyphae‐inducing FBS YPD medium. Untreated cells displayed significant hyphal growth within 3 h, whereas cells treated with Honokiol at MIC levels exhibited only minor morphological alterations, with no filamentous structures. By targeting hyphal development, Honokiol disrupts a critical aspect of *C. albicans* pathogenesis, reducing its ability to invade host tissues and establish infection. Our results further demonstrated that Honokiol inhibited mature biofilms formation, as demonstrated by reducing their metabolic activity by 78.3% in clinical isolates and 72% in the ATCC strain at MIC. A previous study reported that the Honokiol at the concentration of MIC inhibited more than 90% biofilm formation in the early phase and the developmental phase (Sun et al. 2015). Biofilm formation in *C. albicans* is a major clinical challenge, as biofilms are inherently resistant to antifungal treatments (Fan et al. 2022). CLSM images visually confirmed biofilm eradication post‐Honokiol treatment, further supporting these findings. Biofilm disruption is particularly noteworthy, as it represents a critical therapeutic target for preventing persistent infections and ensuring effective fungal eradication. These results suggest that Honokiol can be a valuable agent in addressing biofilm‐associated infections, especially in oral and endodontic environments. Further investigation into Honokiol's mechanism of action revealed its ability to compromise cell membrane integrity. SEM images indicate that Honokiol disrupts the fungal cell membrane. Untreated cells maintained their characteristic oval shape with smooth surfaces, whereas Honokiol‐treated cells exhibited significant deformation. Treated cells displayed convoluted and irregular surfaces, with deep furrows, wrinkles, and the presence of lytic material in vesicular form. These morphological changes confirm that Honokiol exerts a destructive effect on fungal cells, disrupting their structural integrity and viability.

While the study presents promising in vitro results clearly demonstrating the antifungal effect of Honokiol, it does have some limitations. Lack of in vivo validation using an animal model is a major limitation. Our experiments on the 24‐h exposure of dentine‐attached biofilm to Honokiol may not adequately represent the complex, long‐term reality of the oral environment. Microbial biofilms, which include fungi, develop, and persist for several weeks or longer, are continuously challenged by antimicrobial factors, both host‐derived and exogenous.

The findings of this study have significant clinical implications. Honokiol's ability to inhibit biofilm formation, hyphae development, and ergosterol biosynthesis address multiple aspects of *C. albicans* pathogenicity, making it a versatile antifungal agent. Its synergistic interaction with Fluconazole provides an opportunity to enhance the effect of existing treatments, potentially overcoming resistance in challenging infections. Additionally, its effect in disrupting biofilms and compromising cell membrane integrity makes it particularly valuable for treating persistent and recurrent fungal infections.

## 5 Conclusion

Honokiol demonstrates significant antifungal activity against *C. albicans*, as it targets multiple pathways critical for fungal survival and virulence. Its ability to disrupt biofilms, inhibit hyphae formation, and compromise ergosterol biosynthesis underscores its potential as a multifaceted antifungal agent. The synergistic effects with Fluconazole further enhance its therapeutic value, offering a promising strategy to combat antifungal resistance and improve treatment outcomes. These findings warrant further investigation into the clinical applications of Honokiol, including in vivo studies and the development of combination therapies to optimize its effect in managing oral fungal infections.

## Author Contributions

**Maribasappa Karched:** conceptualization, funds acquisition, study design, experimentation, data analysis and interpretation, manuscript drafting, and final approval of the manuscript for submission. **Mohammad Irshad:** experimentation, data analysis and interpretation, manuscript drafting, and final approval of the manuscript for submission. **Jawad M. Behbehani:** study design, data analysis and interpretation, manuscript drafting, and final approval of the manuscript for submission.

## Ethics Statement

This study was conducted in accordance with the Declaration of Helsinki and approved by the Institutional Ethical Committee, Health Sciences Center, Kuwait University (Ref: VDR/EC/2347).

## Conflicts of Interest

The authors declare no conflicts of interest.

## Data Availability

The data that support the findings of this study are available from the corresponding author upon reasonable request.
